# 21 Untargeted Metabolomics Analysis: A Candidate Biomarker and Prognostic Tool in Burn Injury

**DOI:** 10.1093/jbcr/irae036.021

**Published:** 2024-04-17

**Authors:** Burook Misganaw, Desiree Pinto, Tuan D Le, Anthony Pusateri, Arti Gautam, Lauren T Moffatt, Jeffrey W Shupp, Rasha Hammamieh

**Affiliations:** Walter Reed Army Institute of Research, Silver Spring, Maryland; MedStar Washington Hospital Center, Washington, DC; MedStar Washington Hospital Center, Grand Prairie, Texas; Naval Medical Research Unit San Antonio, JBSA-Fort Sam Houston, San Antonio, Texas; Firefighters’ Burn and Surgical Research Laboratory, Washington, DC; Georgetown University School of Medicine, Washington, DC; Walter Reed Army Institute of Research, Silver Spring, Maryland; MedStar Washington Hospital Center, Washington, DC; MedStar Washington Hospital Center, Grand Prairie, Texas; Naval Medical Research Unit San Antonio, JBSA-Fort Sam Houston, San Antonio, Texas; Firefighters’ Burn and Surgical Research Laboratory, Washington, DC; Georgetown University School of Medicine, Washington, DC; Walter Reed Army Institute of Research, Silver Spring, Maryland; MedStar Washington Hospital Center, Washington, DC; MedStar Washington Hospital Center, Grand Prairie, Texas; Naval Medical Research Unit San Antonio, JBSA-Fort Sam Houston, San Antonio, Texas; Firefighters’ Burn and Surgical Research Laboratory, Washington, DC; Georgetown University School of Medicine, Washington, DC; Walter Reed Army Institute of Research, Silver Spring, Maryland; MedStar Washington Hospital Center, Washington, DC; MedStar Washington Hospital Center, Grand Prairie, Texas; Naval Medical Research Unit San Antonio, JBSA-Fort Sam Houston, San Antonio, Texas; Firefighters’ Burn and Surgical Research Laboratory, Washington, DC; Georgetown University School of Medicine, Washington, DC; Walter Reed Army Institute of Research, Silver Spring, Maryland; MedStar Washington Hospital Center, Washington, DC; MedStar Washington Hospital Center, Grand Prairie, Texas; Naval Medical Research Unit San Antonio, JBSA-Fort Sam Houston, San Antonio, Texas; Firefighters’ Burn and Surgical Research Laboratory, Washington, DC; Georgetown University School of Medicine, Washington, DC; Walter Reed Army Institute of Research, Silver Spring, Maryland; MedStar Washington Hospital Center, Washington, DC; MedStar Washington Hospital Center, Grand Prairie, Texas; Naval Medical Research Unit San Antonio, JBSA-Fort Sam Houston, San Antonio, Texas; Firefighters’ Burn and Surgical Research Laboratory, Washington, DC; Georgetown University School of Medicine, Washington, DC; Walter Reed Army Institute of Research, Silver Spring, Maryland; MedStar Washington Hospital Center, Washington, DC; MedStar Washington Hospital Center, Grand Prairie, Texas; Naval Medical Research Unit San Antonio, JBSA-Fort Sam Houston, San Antonio, Texas; Firefighters’ Burn and Surgical Research Laboratory, Washington, DC; Georgetown University School of Medicine, Washington, DC; Walter Reed Army Institute of Research, Silver Spring, Maryland; MedStar Washington Hospital Center, Washington, DC; MedStar Washington Hospital Center, Grand Prairie, Texas; Naval Medical Research Unit San Antonio, JBSA-Fort Sam Houston, San Antonio, Texas; Firefighters’ Burn and Surgical Research Laboratory, Washington, DC; Georgetown University School of Medicine, Washington, DC

## Abstract

**Introduction:**

Untargeted metabolomics, an analysis of the molecular layer comprising many smaller molecular weight biomolecules, has emerged as a tool to diagnose and predict outcomes early in disease courses. Existing studies of metabolic changes following burn injury focus on select targeted metabolites using animal models. This study aims to identify metabolic signatures associated with burn severity and clinical outcomes using untargeted metabolomics analysis in patients.

**Methods:**

Burn patients admitted to a regional center over the span of 4 years were prospectively enrolled in this study. Patient demographics, burn injury characteristics, and blood samples were collected at set timepoints from admission until 4 weeks. Mass spectrometry (MS)-based method was used to measure untargeted metabolomics. Comparison of the MS-based measurements of select metabolites with alternate standard clinical assaying was performed to ensure concordance and reliability. A linear regression model was then used to identify metabolites associated with bun severity (TBSA < 20% and ≥ 20%). Age, gender, BMI, presence of inhalation injury, administration of propofol and supplemental nutrition were added as covariates to control for confounding effects.

**Results:**

Complete metabolome datasets were collected for 58 patients from a total of 115 patients reviewed. The sample had a median TBSA of 18.3%, median age of 38.5 and 27.6% had concomitant inhalation injury. A total of 925 metabolites, including 335 lipids, 183 amino acids, 146 xenobiotics, 24 carbohydrates, and 23 peptides were analyzed. MS-based measurements compared with alternate standard clinical assaying resulted in a high level of concordance (R ~ 0.8). The first two principal components (account for 22% of total variance) show the main drivers of global metabolomics variation are time since admission and severity of injury. At admission, a significant elevation of xenobiotics, amino acids, nucleotide, and lipids related metabolites was observed among severely burned patients (TBSA ≥ 20%). Longitudinal analysis reveal that xenobiotic related metabolites are significantly elevated at admission but are depleted overtime. An abundance of select metabolites at admission showed an excellent ability to predict 30-day mortality (AUROC ~ 90%).

**Conclusions:**

Patterns of metabolic alterations were associated with severe burn injury (TBSA ≥ 20%). Distinct classes of metabolic profiles change in a time dependent manner. Therefore, metabolic profiling has the potential to be used as early indicators of disease severity and progression.

**Applicability of Research to Practice:**

Identifying and utilizing early indicators of disease severity and outcomes before the onset of clinical signs is crucial for burn patients. Metabolomic changes is a prime candidate for the development of these prognostic and biomarker panels.

